# Intravascular lithotripsy modulates calcification without affecting lipid plaque contents *in vivo*: insights from near-infrared spectroscopy and intravascular ultrasound imaging

**DOI:** 10.1093/ehjcr/ytae023

**Published:** 2024-01-12

**Authors:** Issei Ota, Yu Kataoka, Teruo Noguchi

**Affiliations:** Department of Cardiovascular Medicine, National Cerebral and Cardiovascular Center, Suita, Japan; Department of Cardiovascular Medicine, National Cerebral and Cardiovascular Center, Suita, Japan; Department of Cardiovascular Medicine, National Cerebral and Cardiovascular Center, Suita, Japan

A 77-year-old gentleman presented with angina pectoris. Coronary angiography revealed a calcified intermediate stenosis at the middle segment of the left anterior descending artery (fractional flow reserve [FFR] = 0.67, *Panel A*, Supplementary material online, *Video S1*). Near-infrared spectroscopy (NIRS) and intravascular ultrasound (IVUS) imaging (Dualpro™, Infraredx, Bedford, MA, USA) were conducted to strategize PCI procedure. Severe circumferential calcification was observed at the target lesion by IVUS imaging (*Panel B*, Supplementary material online, *Video S2*). Furthermore, NIRS imaging revealed that maxLCBI_4mm_ at this lesion was 767 (*Panel C*). Since maxLCBI_4mm_ ≥ 500 has been shown to reflect a large lipid core plaque causing periprocedural myocardial infarction after PCI, this lesion was considered to harbour a substantial amount of lipidic plaque materials associated with periprocedural risks. Therefore, intravascular lithotripsy (IVL) was performed by using 3.0 mm IVL balloon (Shockwave Medical, Santa Clara, CA, USA, *Panels D*, *E*, and *F*). The NIRS/IVUS imaging after IVL elucidated multiple fractures at the surface of circumferential calcification with a small reduction of maxLCBI_4mm_ (=716) (*Panels G* and *H*, Supplementary material online, *Video S3*). Everolimus-eluting stent (XIENCE™, Abbott Vascular, Santa Clara, CA, USA) was successfully implanted without any procedural complications (see Supplementary material online, *Video S4*).

**Figure:**
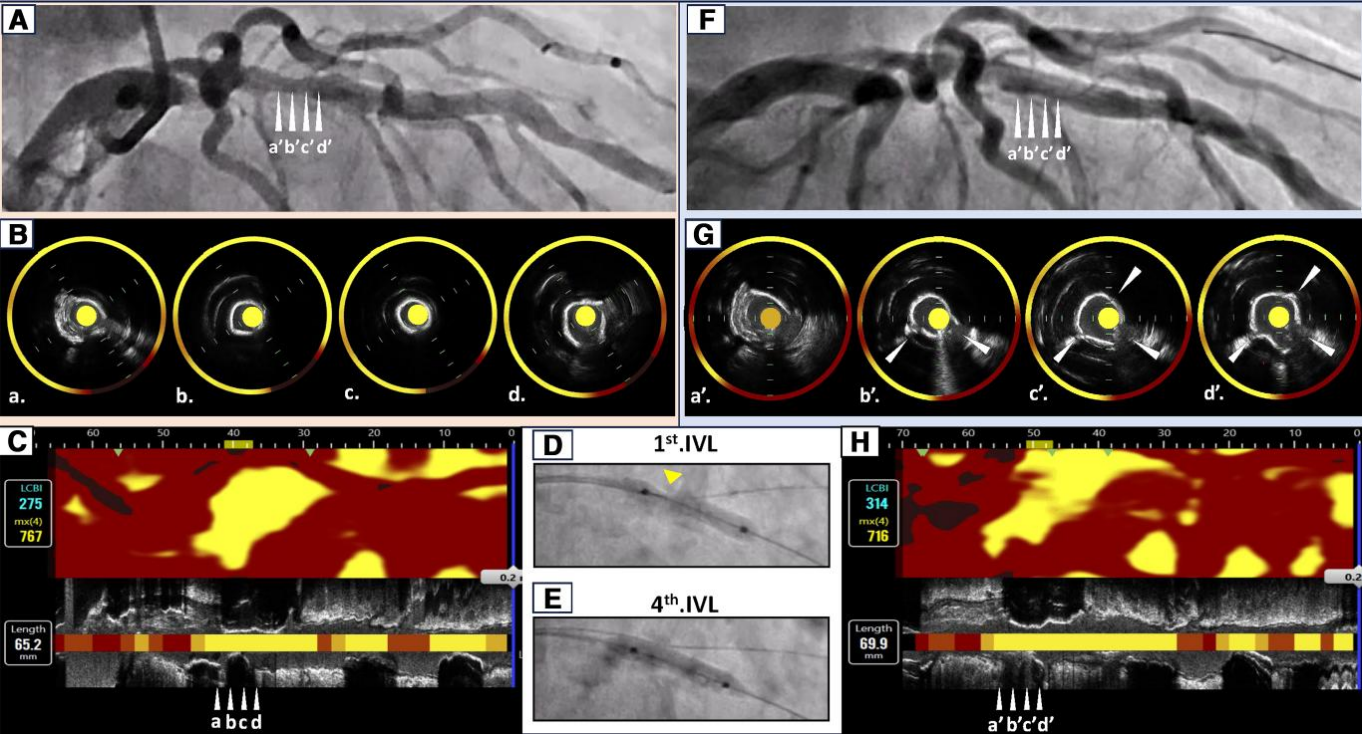


Fracturing effects of Shockwaves are generally observed at calcification tissues without negatively affecting soft tissues. Multi-modality imaging provides mechanistic insights into how IVL modulates calcific and lipidic plaque tissues *in vivo*. In our case, multiple fractures of calcification with small reduction of lipidic contents were observed. These IVL-related effects may result in mitigating risks of worsening coronary flow and periprocedural myocardial infarction which other debulking devices could cause.

## Supplementary material

Supplementary material is available at *European Heart Journal – Case Reports* online.

**Consent:** Written informed consent was obtained from the patient in accordance with COPE guidelines.

**Funding:** Nothing to declare.

## Data availability

No data were generated or analysed for or in support of this paper.

## Supplementary Material

ytae023_Supplementary_Data

